# Why genomics researchers are sometimes morally required to hunt for secondary findings

**DOI:** 10.1186/s12910-020-0449-8

**Published:** 2020-01-31

**Authors:** Julian J. Koplin, Julian Savulescu, Danya F. Vears

**Affiliations:** 1 Biomedical Ethics Research Group, Murdoch Children’s Research Institute, Melbourne, Australia; 2 Melbourne Law School, University of Melbourne, Melbourne, Australia; 3 Faculty of Philosophy, Oxford Uehiro Centre for Practical Ethics, Oxford University, Oxford, UK

**Keywords:** Genomics, Research ethics, Incidental findings, Secondary findings, Duty to rescue

## Abstract

**Background:**

Genomic research can reveal ‘unsolicited’ or ‘incidental’ findings that are of potential health or reproductive significance to participants. It is widely thought that researchers have a moral obligation, grounded in the duty of easy rescue, to return certain kinds of unsolicited findings to research participants. It is less widely thought that researchers have a moral obligation to actively look for health-related findings (for example, by conducting additional analyses to search for findings outside the scope of the research question).

**Main text:**

This paper examines whether there is a moral obligation, grounded in the duty of easy rescue, to actively hunt for genomic secondary findings. We begin by showing how the duty to disclose individual research findings can be grounded in the duty of easy rescue. Next, we describe a parallel moral duty, also grounded in the duty of easy rescue, to actively hunt for such information. We then consider six possible objections to our argument, each of which we find unsuccessful. Some of these objections provide reason to limit the *scope* of the duty to look for secondary findings, but none provide reason to reject this duty outright.

**Conclusions:**

We argue that under a certain range of circumstances, researchers are morally required to hunt for these kinds of secondary findings. Although these circumstances may not currently obtain, genomic researchers will likely acquire an obligation to hunt for secondary findings as the field of genomics continues to evolve.

## Background

Consider the following hypothetical scenario:

*Joseph is a 30 year old with focal segmental glomerulosclerosis (FSGS), a progressive condition that affects the kidneys and eventually leads to renal failure. Although Joseph’s condition is suspected to have a genetic aetiology, genetic testing performed in the clinical context to date has not identified the gene responsible. The clinical geneticist has offered Joseph the option of participating in a research study that is performing genomic sequencing on patient-participants who have FSGS but in whom a genetic cause has not yet been identified. In discussions with the researcher associated with the project, Joseph learns that during the analysis of his genomic data, as well as identifying the cause of his FSGS, the researchers may also see variations in other genes that are unrelated to his renal condition. These variants, often referred to as ‘unsolicited’ or ‘incidental’ findings, may show that Joseph is at risk of any number of other genetic diseases. This can range from neurofibromatosis to Alzheimer’s disease to being a healthy carrier of cystic fibrosis. Joseph is asked whether he wants to be informed about these if they happen to be identified inadvertently during the analysis, to which he consents. Joseph – who, it turns out, is a molecular geneticist – also has an additional request. He asks the researcher if they are able to actively search for an additional set list of disease-causing genes. For example, Joseph would like the researcher to check for variants associated with hypertrophic cardiomyopathy, amongst other things, which would require the researcher to conduct additional analysis. The researcher explains that performing the additional analysis required to search for these ‘secondary findings’ is outside the scope of the research and therefore they will only return disease-causing variants that are identified inadvertently during the course of the analysis.*

As this scenario suggests, participants in genomic research sometimes receive valuable information about genetic variations of which they may otherwise have remained ignorant. However, whether a particular variation comes to light often depends on whether researchers stumble upon this variation during the course of research. In the research setting, the term ‘unsolicited findings’ refers to genetic variations that are of potential health or reproductive significance to a research participant, and are identified inadvertently when genomic sequencing is performed to find the genetic basis for their existing genetic condition [1]. This is in contrast to the term ‘secondary findings’, which refers to variants in disease-causing genes that are secondary to the research question yet are actively searched for by the laboratory scientists conducting the study.

It is increasingly thought that clinicians and researchers ought to disclose at least *some* kinds of unsolicited findings to participants of clinical research studies, although the obligations may be more tenuous for biobank participants and secondary use of data. Specifically, there is an emerging consensus that clinicians and researchers have a moral obligation to return *at least* individual genetic research results that are of high clinical importance – i.e., sufficiently likely to result in a serious genetic condition – and actionable – i.e., where something can be done to prevent or treat the disease [2–4]. In Australia (where the authors of this paper are based), the National Health and Medical Research Council holds that some unsolicited findings must be returned to participants, depending on their degree of analytic validity, clinical utility, and importance to the health of participants or their immediate family [5]. Here and elsewhere, researchers are widely believed to have strong moral reasons to disclose genomic information that could promote participants’ wellbeing.

It is less widely believed that researchers should actively hunt for such information in the course of their research. To the contrary, many commentators explicitly argue that researchers have no moral obligation to actively hunt for secondary findings in the context of genomic research [6–9]. This accords with the guidelines of the Australian National Health and Medical Research Council, which hold that researchers do not have an obligation to look at findings outside the scope of their research question [5].

In contrast, in the context of genomic *medicine,* the idea of a duty to hunt for secondary findings has received some support*.* The American College of Medical Genetics and Genomics (ACMG) has recommended that laboratories actively search for a set list of variants that indicate an increased risk for hereditary cancers or cardiac conditions, regardless of the genetic condition for which genomic sequencing is being performed [10]. Because these variants are thought to have clear clinical utility and actionability, the ACMG considers that early detection will lead to significant health benefits for individuals (and potentially also other family members). However, the ACMG recommendation has proven controversial, in part because many of its critics reject the idea of a duty to look [11, 12]. Even in the clinical context, the idea of a moral obligation to actively look for secondary findings has met resistance.

This is not to claim that there is a complete consensus against hunting for secondary findings in research. Some recent scholarship defends active hunting in some situations [13, 14], and some institutional review boards have recommended screening for variants on the ACMG list (even though the ACMG guidelines were intended only for the clinical realm.) However, this practice remains uncommon, particularly outside of the United States.

In this paper, we argue that if it is sometimes appropriate for researchers to disclose unsolicited findings, then it will sometimes also be appropriate for researchers to actively hunt for this information. If we recognise a duty to disclose secondary findings, we implicitly acknowledge that researchers sometimes ought to promote the interests of research participants *even if* doing so imposes some costs on the researchers. But if it is sometimes appropriate to promote participants’ wellbeing by disclosing unsolicited findings, then – at least in principle – it is presumably also sometimes appropriate to promote participants’ wellbeing by seeking such information out.

We proceed as follows. First, we first show why a moral duty to disclose (some categories of) unsolicited findings seemingly entails a moral duty to actively look for secondary findings. We then consider several objections to this argument. While we reject most of these objections, we concede that researchers may not *currently* have a duty to screen for secondary findings, given the magnitude of the costs of doing so and the uncertainty of the benefits. Importantly, however, this objection – unlike the other, more commonly-presented arguments – leaves open the possibility that we should hunt for secondary findings once certain conditions are met. Even if researchers do not currently have a duty to search for secondary findings, they are likely to acquire such a duty in the future.

Before we proceed, there are two aspects of our argument that we would like to clarify. First, we acknowledge that there is an important question that we do not address in this paper: whether patients/participants should be able to opt out of receiving (certain types of) findings, or – conversely – whether certain kinds of information should be reported regardless of whether patient/participants give valid consent [15]. Much has already been written on this topic. For the purpose of this paper, it is enough to specify that *if* research participants ought to be allowed to opt out of receiving secondary findings, then the duty to search for secondary findings is contingent on participant consent.

Second, for the sake of simplicity, we speak in this paper about *researchers’* duties of easy rescue. Our main focus, however, is the moral obligations of research institutions rather than individual researchers. It is research institutions, not individual researchers, who decide and enforce rules on whether researchers should return incidental findings or hunt for secondary findings. We are interested in what these rules should be. In other words, we are interested in what is sometimes called the *institutional* duty of easy rescue – a moral duty, held by institutions, to facilitate easy rescues [16, 17]. This strikes us as the correct level for the discussion. The debate around incidental and secondary findings centres on the rules that institutions should adopt or professional bodies such as the ACMG should recommend, not the actions that individual researchers should take.

## Main text

### Why the duty to disclose individual research findings implies a duty to hunt for them

The duty to disclose clinically important and actionable unsolicited findings is often (though not always) grounded in the duty of easy rescue. This view occurs throughout the ethics literature on genomic research [2, 3, 18–20]. This intuition also seems to shape the thinking of at least some ethics committees; a recent quantitative study of the perspectives of 796 IRB members in the United States found that something akin to the duty of easy rescue was the most common rationale for returning genetic incidental findings [21].

What, precisely, do we mean by the ‘duty to rescue’? We have in mind a moderate version of the duty of easy rescue, such as the following:

*If the cost … to someone of performing an action X … is sufficiently small to be reasonably bearable, and the resulting [or expected] benefit to other people (or harm that is prevented) is large relative to the cost, then the agent ought to do X* [22].

This version of the duty of easy rescue includes two conditions. First, the costs to the rescuer need to be proportionally much smaller than the benefits to the beneficiary. Second, the costs borne by the rescuer need to be reasonably bearable. We understand ‘reasonably bearable’ in absolute terms; the duty of easy rescue cannot require us to make major sacrifices, no matter how much good we might thereby achieve.

We understand benefits in terms of expected utility (taking into account both the magnitude of the potential benefits and the probability they will be realised.) Accordingly, as the chance that one will benefit somebody decreases, so do the expected benefits of attempting the rescue. We understand the duty of easy rescue to apply not only when we would save somebody directly, but also when our actions are merely part of the process by which somebody is saved. For example, we think the duty of easy rescue requires us to call an ambulance for the victim of a hit-and-run – even though it is the paramedics, and not us, that would ultimately perform the rescue. We also understand the duty of easy rescue to apply not only to scenarios where we could save a life, but also to scenarios where we can mitigate suffering. We ought to call an ambulance not only for people in mortal peril, but also for those who are at risk of losing a limb, experiencing long-term disability, or enduring intense pain that could be ameliorated. What matters in cases of ‘easy rescue’ is expected utility (where ‘utility’ is understood as wellbeing.)

Admittedly, this formulation of the duty to rescue is different from other formulations of the duty that have been discussed in the genomics literature, some of which are limited to *imminent* risks of *extreme* harm and/or death [14, 15]. Although our version of the duty to rescue is broader than these (extremely narrow) formulations, we think it nonetheless captures a highly plausible moral view – i.e., that we are morally required to help others when the harm we might avert is sufficiently great, and the costs to us are sufficiently small. Morality, as opposed to self-interest or prudence, is about promoting the well-being or autonomy of others, as opposed to one’s own. A duty of easy rescue embodies a *minimal* morality.

Our formulation of the duty to rescue is consistent with the view that researchers should disclose (at least some categories of) unsolicited findings. It specifies two conditions for the disclosure of these findings. First, the expected benefit of disclosing this information must greatly outweigh the costs to researchers. Second, the costs to researchers must be reasonably bearable, *regardless* of how weighty the moral gains would otherwise be. If both conditions are met – which would presumably be the case for many serious and clinically actionable findings – then researchers ought to disclose unsolicited findings to research participants.

Less obviously, this formulation of the duty of easy rescue lends support to the view that researchers have a defeasible moral duty to hunt for the same kinds of findings that they ought to disclose. If hunting for secondary findings is expected to provide significant benefits to research participants, and if the costs to researchers are small in comparison to these benefits and reasonably bearable, then – according to (our formulation of) the duty of easy rescue – researchers ought to do so. Seen this way, our moral reasons for hunting for secondary findings parallel our moral reasons for disclosing them. If researchers sometimes have a rescue-based moral obligation to disclose unsolicited findings, then presumably they also sometimes have a rescue-based moral obligation to seek this information out.

We are not here taking a specific stance on the set of variants predisposing to particular conditions that ought to be returned. As mentioned previously, the ACMG has developed a “minimum list” of genes they believe should be actively searched for in all patients undergoing clinical genomic sequencing, based on expert opinion that variants in these genes will cause disease [10, 23]. However, this determination is based on the expression of disease in individuals already affected with these conditions; we have little information about the potential for these variants to cause disease in currently unaffected individuals. Some argue that the medical benefits to returning this information are uncertain, and potentially meagre [11, 24]. Returning such information might even be harmful if healthy participants access additional screening or undertake unnecessary and potentially harmful prophylactic measures (for example, surgery to remove ovaries in a woman with a variant in *BRCA2* that is initially classified as likely to be pathogenic but later reclassified as a benign variant). It is possible, then, that reporting certain incidental or secondary findings might cause more harm than good.

For the sake of our argument, we can deal with these issues by making the duty to search for (a certain set of) secondary findings contingent on whether these sorts of findings ought to be disclosed in the first place. If a country, group, or institution believes that we lack sufficient evidence about whether a particular genetic variant is pathogenic, then this variant should neither be reported nor actively hunted for. If, however, a country or group *does* think that reporting a particular (set of) variant(s) would yield sufficient medical benefit to disclose them, then – subject to cost constraints – this (set of) variant(s) should be actively looked for in research participants. We are arguing, in other words, for symmetry; we propose that the kinds of genetic variants that ought to be reported also ought to be sought out.

Even if it turns out that researchers are not *currently* required to either report or search for genetic findings in research participants, this will likely change over time. Scientific understanding of individual genetic results (and associated variants that modify whether or not they are in fact disease-causing) is continually increasing. This improved knowledge can be used to refine the list of secondary findings that are worth testing for. It will also help us predict who requires additional investigations, such as MRIs or surveillance, and who does not. This will reduce the impact of unnecessary investigations on the healthcare system, the potential harms of unnecessary interventions, and any unwarranted patient anxiety based on inaccurate predictions of risk. Accordingly, even if genomic researchers do not currently have a duty to report and hunt for genetic variants such as those on the ACMG list, they will likely acquire such an obligation in the future.

We have offered a brief defence of the view that genomic researchers sometimes have a moral duty, grounded in the duty of easy rescue, to actively hunt for secondary findings. Over the remainder of this paper we consider the main objections that have been or could be levelled against it.

### Objection 1: rescues that result from hunting for unsolicited findings are not genuinely easy rescues

Our argument so far relies on an analogy between disclosing incidental findings and hunting for secondary findings. However, there is one obvious respect in which these practices differ: the costs of hunting for secondary findings are likely to be much higher than the costs of disclosing any incidental findings one happens to turn up. Accordingly, at least at present, it might be the case that the duty of easy rescue only requires the disclosure of incidental findings, and not that one seeks this information out.

Recall that the duty of easy rescue applies only if the costs borne by rescuers are both greatly outweighed by the benefits *and* reasonably bearable in absolute terms. For research institutions, a ‘reasonably bearable’ cost is one that does not seriously jeopardise its ability to fulfil its primary purpose (e.g., to produce generalisable medical knowledge.) The duty of easy rescue only applies if both conditions are met.

There are two key reasons why hunting for secondary findings might run afoul of these conditions. First, even though participants’ genomic sequence data would already exist, searching this data for secondary findings requires laboratories to perform significant additional analysis and interpretation. Second, in cases where genetic counsellors are employed to return individual research results, searching for secondary findings would presumably require greater engagement of genetic counsellors; the genetic counselling resources required for a study depends on the number of participants in the study and the incidence secondary findings.

What this means is that the costs of hunting for secondary findings are higher (both in relative and absolute terms) than merely disclosing whatever incidental findings one happens to stumble upon. Accordingly, it might be the case that searching for secondary findings does not currently fall within the scope of the duty of easy rescue. In some cases, a research institution might have a duty to disclose potentially harmful genetic variants, but not to seek these variants out. However, this does not rule out the idea of a duty to hunt for secondary findings *tout court.* Where institutions can reasonably bear the cost, they ought to seek out (an appropriately tailored list of) secondary findings. Moreover, as better processes are developed, the costs associated with the additional analysis and interpretation will decrease, as will the time taken to perform the secondary analysis. While we concede that searching for secondary findings does not constitute an easy rescue at this point in time, this only pertains to the current state of knowledge and is likely to change in the not-too-distant future.

We think it would be useful to conduct further research on the costs and benefits of hunting for specific lists of clinically significant secondary findings, such as the ACMG list. One option is to actively look for findings on this list in the context of a research project, where the medical, financial and psychosocial outcomes of identifying them are systematically followed-up on. Once we better understand the costs and benefits of hunting for secondary findings, we will be better able to determine whether genomic researchers have a moral obligation, grounded in the duty of easy rescue, to actively seek these findings out.

Our arguments in this section are not entirely novel. Gliwa and Berkman, for example, have previously argued that researchers may acquire a duty to look for genetic incidental findings once a) the medical benefits that could be achieved by doing so increase, b) the burdens it poses on researchers decrease, and provided that c) participants are unlikely to receive this beneficial information from other sources (for example, via physicians or direct-to-consumer genetic testing companies) [13]. We consider our arguments complementary to Gliwa and Berkman’s analysis. Where Gliwa and Berkman ground their arguments in (a controversial understanding of) researchers’ ancillary care obligations, we ground ours in the duty of easy rescue. Since the duty of easy rescue is a widely-accepted moral obligation – and, moreover, is commonly cited as a reason to disclose incidental findings – we think this duty can provide an especially strong reason to hunt for secondary findings.

### Objection 2: the duty to disclose unsolicited findings is properly grounded in researchers’ ancillary care obligations, not their duties to rescue

We have drawn on the duty of easy rescue to argue that researchers have moral duties not only to disclose unsolicited findings, but also to hunt for secondary findings. However, there is another way of understanding the duty to disclose unsolicited findings: as a specific facet of researchers’ ancillary care obligations. There is an important difference between these two ways of understanding researchers’ duty to disclose. With some exceptions - such as Gliwa and Berkman, in the paper discussed above [13] - those who adopt the ancillary care framework hold that it only provides moral reason to *disclose* unsolicited findings that are stumbled upon while conducting the research project. Unlike the duty of easy rescue, the ancillary care framework need not be understood to provide moral reason to actively hunt for secondary findings.

Why is this the case? Under (at least some versions of) the ancillary care framework, medical researchers are thought to have a special obligation to help research participants with some specific kinds of medical needs: those that come to light as a result of researchers learning information about participants that they would not otherwise have had access to [8, 25, 26]. For example, on Richardson’s partial entrustment model for ancillary care, researchers have a moral duty to disclose unsolicited findings as a kind of compensation for the relaxation of participants’ privacy rights. Because this breach of privacy provides the impetus for disclosing unsolicited findings, researchers’ ancillary care obligations are limited to addressing needs that happen to be discovered over the course of the research (as originally designed). The ancillary care framework (so understood) does not require researchers to screen for findings not directly relevant to the research question. Indeed, on Richardson’s model, expanding the scope of screening beyond what is required for the study is usually considered prima facie inappropriate, as doing so would deepen the breach of participants’ privacy [25]. Those who are convinced by the ancillary care framework might think that the duty to look should be rejected on these grounds.

However, this objection is a nonstarter, for the simple reason that the ancillary care framework cannot – and is not intended to – capture the full range of researchers’ moral obligations. Instead, the ancillary care framework is intended to capture only one specific set of moral obligations - those connected to the distinctive relationship that exists between researchers and participants [26]. Accordingly, even if the ancillary care framework captures the full range of researchers’ moral obligations *qua* their role as researchers, researchers might have additional moral duties that are connected to the other roles they fulfil. One such duty is the duty of easy rescue, which falls on *all* moral agents – researchers included. Even if the ancillary care framework does not support a duty to search for secondary findings, researchers may nonetheless have a rescue-based obligation to search for secondary findings.

Admittedly, grounding obligations to research participants in the institutional duty of easy rescue can have counter-intuitive implications. Imagine a scenario where an individual downloads their genomic data from a 3rd party genetic ancestry service, then approaches a genomic researcher to screen this data for secondary findings. (They are not part of the researcher’s study.) Since the duty of easy rescue is owed to all persons, it would seem to commit researchers (and their institutions) to searching for findings in the data of people who are not participants in their research. This conclusion might seem implausible, or at least surprising.

We think that researchers do have the same duty of easy rescue as any other person. In theory, if it were possible to provide potentially life-saving information at no or negligible cost, then researchers ought to do so. This is a straightforward upshot of the idea that persons in general have a duty of easy rescue. Researchers (and their institutions) have the same moral duty as any other citizen to save others’ lives if they can easily do so.

In practice, however, there are several practical reasons why the duty of easy rescue does not require researchers to screen the data of people who aren’t research participants. First, offering screening services to persons in general would presumably impose much greater costs on research institutions than if screening were limited to participants. Second, it might be that researchers (and institutions) owe additional duties to their participants (such as duties of reciprocity) that provide additional reasons to help research participants over people in general. Third, since research institutions cannot control the quality of the data provided by online genomic sequencing companies, this data might be more difficult to interpret – and the benefits of doing so less concrete –than in the case of research participants’ data. (In fact, many online DNA tests only provide data for particular markers in one’s DNA, not actual sequence data, meaning that searching for the types of secondary findings we are referring to is impossible.) Fourth, in cases where researchers do not return raw genomic data to participants, they are uniquely positioned to screen this data. Somebody who already has access to their raw data could seek analysis and interpretation elsewhere; research participants often cannot. Accordingly, while the duty of easy rescue is a general duty, researchers have stronger reasons to perform genetic screening for research participants than members of the general public.

### Objection 3: duties of easy rescue do not require us to seek out opportunities to perform easy rescues

It is sometimes argued that duties of easy rescue do not entail that we are required to *seek out* opportunities to perform easy rescues. For example, Ulrich argues that duties of easy rescue apply *only* when one finds oneself in a situation to perform an easy rescue; they “d [o] not compel anyone to search out harm that one may be able to alleviate” [18]. Accordingly, one might think that researchers have a duty to rescue, but not (what might be called) a *duty to look.*

This view is not unique to the literature on unsolicited findings. Much philosophical work on the duty of easy rescue likewise assumes that we do not have a moral obligation to actively seek out rescue opportunities [27]. If this view is correct, we can consistently recognise a moral obligation to disclose unsolicited findings identified inadvertently while denying any moral obligation to actively seek them out.

We do not think the duty of easy rescue is so easily divorced from the duty to seek out rescue opportunities. Consider the following two scenarios:

**Certain opportunity for a possible rescue**

You are walking over a bridge that spans a fast-flowing river when you hear somebody calling for help. Looking over the balustrade, you notice somebody floundering in the fast-moving current.

A life preserver hangs nearby. You could attempt to throw it to the victim below. Unfortunately, the current is rapidly carrying them downstream, and you estimate that there is only a 50% chance the life preserver will reach them.

Anybody in this situation should throw the life preserver. We assume this view would be uncontroversial. The costs of throwing the life preserver are negligible, whereas the potential benefits are enormous. People have a moral obligation to *attempt* easy rescues, even if success is not guaranteed.

**Possible opportunity for a certain rescue**

You are walking near a slow-flowing river when you hear somebody calling for help. A life preserver hangs on a nearby bridge. You could walk over and check if anybody needs assistance. If they are, you are confident that you could throw the life preserver to anybody floundering in the sluggish waters below.

There have been several recent drownings near this very bridge, but also several occasions where local troublemakers sought to alarm nearby pedestrians by calling for help while standing on the riverbank. Given this history, you estimate there is a 50% chance that somebody is in genuine peril. It would take only a short walk to the bridge to find out either way.

The morally salient features of this second scenario track the morally salient features of the first. The costs of making an unnecessary journey to the bridge are negligible, whereas the potential benefits are enormous. Anybody in this situation should walk to the bridge to check if anybody needs rescuing. This is because people have a moral obligation to seek out rescue opportunities when a) it is easy to do so, and b) the harms that might be averted are significant. They have a moral obligation to do so even if they are not guaranteed to find anybody in need of rescuing.

In both scenarios, there is a 50% chance that one could save a life at little cost to one’s self. In both scenarios, one is morally required to act. It does not make any obvious moral difference whether one is considering performing an easy rescue or looking for an opportunity to perform an easy rescue.

One might object that there is some reason to believe there is a person in need in the second scenario, while there is no reason to believe the research participant is in need (in the sense that they would benefit from having researchers hunt for secondary findings). But this is false. Statistically, everyone is at risk of having a disposition to disease. For example, based on population prevalence estimates, the chance of having a genetic variant for familial hypercholesterolemia (which causes coronary artery disease) is between 0.2–0.5% [23]. This probability is for just one condition. One’s chance of having a mutation on the ACMG’s 56-gene list is much higher; one estimate places the probability at 1.7% [28]. Since each variant on the ACMG list is actionable, highly penetrant, and medically serious, the benefits of receiving this information would often be profound. As our understanding of genomics improves, additional variants will likely be added to such lists, further increasing researchers’ odds of discovering medically beneficial information [13]. Statistically, research participants are at risk of a genetic disposition to disease and so are in need.

It might be thought that a 1.7% probability of saving a life is too low to count as an easy rescue. The probability, however, is not the only relevant factor here; what matters is how the costs we would incur by making the attempt compare to the expected benefits of doing so. If it were possible to push a button that grants a 1 in 60 chance of saving somebody, then we ought to do so; the costs of pushing the button are dwarfed by the expected benefits of the action, even taking into account the low likelihood these benefits would eventuate. While it is not the case now, if screening for a set of secondary findings were as easy as merely pushing a button, then researchers *should* push this button. As the costs of searching for secondary findings decrease (and as the benefits of searching increase), it will become increasingly plausible to think that secondary findings ought to be actively hunted for. What matters in rescue is expected utility for the rescuer and rescuee, which is a function of the expected costs of searching vs the expected benefits of doing so (factoring in both the magnitude of the cost/benefit and the likelihood these costs/benefits will eventuate).

Put formally and fully, an easy rescue is one where the expected cost to the rescuer is small and the expected benefit to the beneficiary is large.

Why, then, is it commonly thought that we are required to perform easy rescues, but not required to seek out easy rescue opportunities? One reason is because the idea of a duty to seek out rescue opportunities sounds highly demanding. If the duty to look for rescue opportunities is interpreted extremely broadly, it might seem that recognising such a duty would commit us to searching, for all of the rest of one’s days, for persons in need of easy rescues – or to go out of our way to monitor any rivers within our general vicinity for persons who happen to be drowning. An open-ended duty to commit one’s life to this cause seems unreasonably onerous [27].

We agree that extremely broad versions of the duty to look are implausibly demanding. However, moderate versions of the duty to look do not give rise to this issue, provided that certain criteria must be met before this duty applies. As such, in line with the moderate duty of easy rescue described above, a moderate duty to look requires one to look for rescue opportunities *only* if (i) the costs of doing so are sufficiently small to be reasonably bearable and (ii) the expected benefit is large relative to the cost (factoring in both the magnitude of the benefit and the likelihood it will eventuate). Understood this way, the duty to look would not require us to disrupt the normal course of our lives unless there is a non-trivial chance that doing so would benefit others.

Consider one final thought experiment:

**Likely opportunity to perform multiple easy rescues**

You are fishing off the shore of the mainland when you hear, over the radio, that a cruise ship has sunk nearby and that many of its passengers are floundering in the waters nearby. You have the necessary equipment and capability to help with the rescue efforts. It is highly likely that if you made a brief journey to the location of the accident, you would encounter several opportunities to perform easy rescues (of people who may otherwise drown). However, in order to do so, you would need to *actively look* for these rescue opportunities; from where the boat sits now, you can see nobody in peril.

People do not have a general moral duty to search the oceans randomly or indefinitely for persons at risk of drowning. However, given the balance of likely costs and benefits, we *do* think that anybody in the above scenario has a clear moral duty to look for persons that they might be able to rescue. Moreover, we think that screening for secondary findings resembles this scenario in some important respects; if every research participant is screened for secondary findings, there is a good chance that the researchers will find opportunities to perform easy rescues. Moderate versions of the ‘duty to look’ are no less plausible than (and should arguably be considered a component of) moderate versions of the duty to rescue.

### Objection 4: rescue obligations should be met via the broader healthcare system

It might be thought that the responsibility to meet research participants’ health needs ultimately lies not with individual researchers, but rather with the broader healthcare system – that it should not be researchers who ‘push the button’ of whole genome analysis, but the health system. For example, Garrett argues that bioethical discussions of unsolicited and secondary findings should not focus on whether individual researchers and/or institutions have opportunities to perform easy rescues. Instead, Garrett recommends focusing on the question of how we can best *collectivize* these rescue obligations – for example, by implementing new forms of targeted or population-wide genomic screening. The argument here is that if we want to promote public health, we should focus our attention on the structure of our broader healthcare system; research institutions, by contrast, should focus their resources on the generation of generalizable medical knowledge [29]. Accordingly, Garrett argues that research protocols should often be designed to minimise the incidence of identifying unsolicited findings.

There are two key ideas here that need to be unpackaged. The first is the assertion that ‘collectivizing’ rescue obligations can help us meet people’s needs more efficiently and equitably. There are many contexts where this is clearly true. For example, we can fight fires more effectively by setting up (and collectively supporting) a trained force of firefighters than by individually seeking to fight any fires we happen to notice [30]. Similarly, it might also be the case that our rescue obligations towards people with undetected genetic conditions would be best met collectively – for example, via public screening programs – rather than by looking for secondary findings in research participants. As Jarvik and colleagues have commented, when seen from a public health perspective, hunting for secondary findings *only in individuals who happen to participate in genomic research* amounts to a strikingly low-yield form of health screening [3]. It is also inequitable, as only those who get enrolled in research and have sequencing will have access to receiving secondary findings.

We agree that it is worth considering whether, or the conditions under which, genomic testing should be offered in a public screening context. Although we do not attempt the task in this paper, we think it is worth investigating whether genomic researchers’ rescue obligations can be ‘collectivized’ in this way.

The second component of Garrett’s argument is more problematic. This is the idea that *because public health needs are ideally managed* via *the healthcare system,* individual research institutions do not have a moral obligation to look for rescue opportunities – even if these health needs are not currently being met. We do not think this is correct. One ought to perform easy rescues (and look for easy opportunities to do so) even if somebody else has a greater responsibility to prevent this harm but is failing to do so. Consider the following thought experiment:

**Negligent lifeguard**

You are walking near the beach when you spot a small child drowning near the shore. A lifeguard is on duty nearby; however, when you point the child out to them, they say they are exhausted from long hours in the sun and are too tired to rescue the child. Given that the lifeguard is failing to perform their duty, you wonder whether you ought to rescue the child instead.

We think that anybody in this situation should rescue the child, even though the lifeguard has a more stringent moral duty than the beachgoer to perform this rescue. Similarly, even if rescue obligations in genomics would ideally be managed by health systems or doctors rather than individual research institutions, this does not relieve these institutions of the moral duty to (look for opportunities to perform) easy rescues when the opportunity to do so arises.

### Objection 5: we only have a moral duty to perform *one-off* easy rescues

A related objection holds that the duty of easy rescue applies *only* to one-off opportunities to rescue others. Rescue opportunities in genomic research are far from one-off. If researchers hunt for secondary findings, they will presumably encounter repeated opportunities to benefit participants by providing them with genomic information that could promote their health. Some commentators have argued that although genomic researchers may have a moral duty to make occasional easy rescues when they stumble upon opportunities to do so, they are not required to (look for opportunities to) perform *frequent* rescues [29].

This objection fails. As per our formulation of the duty of easy rescue, we are morally required to perform multiple easy rescues whenever the costs of doing so are reasonably bearable (and the harm we avert far outweighs the costs we run); having performed one recent rescue does not provide a legitimate excuse for failing to perform another. Consider yet another variant of the drowning child thought experiment:

**Repeated near-drownings**

You are walking by a pond when you spot a toddler at risk of drowning in the shallows. This is the tenth time you have come across this scenario in as many days. These rescues have not been onerous to perform. However, given that you have recently saved several such children – and realising that you might be called upon to rescue more in the future – you wonder whether you actually have a moral duty to save this tenth child.

The frequency of rescue opportunities does not make any obvious moral difference. Given the balance of costs and benefits, anybody in this (granted unusual) scenario should rescue the tenth child. Similarly, genomic researchers should perform easy rescues (and look for opportunities to do so) *even if* they regularly encounter opportunities to do so.

There is a possible problem here. The duty of easy rescue is supposed to be undemanding. However, one might worry that if one is morally required to perform *repeated* easy rescues, one will end up needing to make overwhelming sacrifices to help others in need. Consider *The Life You Can Save*, Peter Singer’s seminal book on charitable giving [31]. Singer argues that if we can easily save the life of a drowning child then we are morally required to do so, even if (say) entering the water would muddy and ruin our new pair of $200 running shoes. By the same token, Singer argues, if we can save a child’s life by making a $200 charitable donation, then we are morally required to do so; the loss of $200 matters much less than the loss of a child’s life. The problem is that there are many people’s lives we could save in this way – more than any one individual can save, regardless of how much they give. Although donating $200 might be easy, making several hundred such donations is not.

It might be the case that we *are* morally required to sacrifice most of our income to charity. The duty of easy rescue, however, is not meant to be so demanding. While the duty of easy rescue is not limited to one-off cases, it *is* limited to cases where the costs of helping others are reasonably bearable. In the context of charitable giving, this might mean that we are morally required to make repeated charitable donations, but not to the extent that we need to sacrifice goals that we find highly important. (In *The Life You Can Save*, Singer proposes some relatively modest targets for charitable giving that might fit this bill: roughly 5% of annual income for the financially comfortable, and somewhat more for the wealthy [31].) In the context of genomic research, the duty of easy rescue – as we have defined it – might require researchers to make repeated sacrifices, but not to the point of jeopardising the success of the research project or the sustainability of the research institution. The duty of easy rescue only recommends searching for secondary findings if a) research institutions can realistically bear the costs of searching for these findings, and b) the benefits to participants greatly outweigh these costs.

### Objection 6: hunting for secondary findings would undermine scientific progress

One might worry that hunting for secondary findings would distract researchers from the central moral purpose of their research: to produce generalisable medical knowledge. If we require researchers to look for reasonable opportunities to perform easy rescues as well as conducting their research, we threaten the efficiency of the research enterprise. It might be thought that resources for research should be dedicated to scientific discovery rather than to promoting participants’ health*, even if* – in line with the duty to rescue – the costs of looking for actionable secondary findings are both reasonably bearable and small in comparison to the expected benefits [3].

The first thing to note is that this objection, as stated, would *also* seem to rule out the disclosure of unsolicited findings. This is because returning individual genomic results that are relevant to the research question *also* requires researchers and their institutions to expend time and effort that could otherwise be devoted to the research project, as does developing the initial plan to manage these findings. If we rule out hunting for secondary findings on the grounds that it is never appropriate for researchers to undermine or delay the production of generalisable medical knowledge, we would presumably also need to rule out the disclosure of unsolicited findings when they are identified. Conversely, if one holds that researchers should disclose (certain kinds of) unsolicited findings, one must also hold that it is sometimes appropriate for medical researchers (and their institutions) to pursue goals other than the production of generalisable medical knowledge.

As described above, much of the bioethical commentary on incidental findings agrees that researchers should disclose at least some categories of unsolicited findings. At the same time, a high proportion of research participants and the general public are keen to receive unsolicited findings [32–35]. These views are incompatible with the view that resources for research should be *exclusively* dedicated to scientific progress.

The second thing to note about this objection is that there is no obvious reason why we should place absolute weight on the production of generalisable medical knowledge. To the contrary, we suspect that many will have strong intuitions that one ought to perform easy rescues – e.g., by actively searching for secondary findings – *even if* by doing so one delays the production of generalisable medical knowledge. Consider the following thought experiment:

**Easy rescue with a slight cost to scientific progress**

A genomics researcher is walking to work when they spot a child drowning in a pond. If they stop to rescue the child, they will not arrive back at work until much later that day. (Among other inconveniences, they would need to shower and change their muddied clothes.) The researcher’s absence would pose a non-trivial inconvenience for the research team and slightly delay progress on the study.

Presumably any researchers in this situation should rescue the child. Accordingly, we cannot rule out the duty to actively hunt for potentially lifesaving secondary findings merely because hunting for these findings would detract from the generation of generalisable medical knowledge.

## Conclusions

This paper has taken an unfamiliar route to a partly familiar destination. We have suggested that the duty of easy rescue does not currently establish a moral obligation for genomic researchers to hunt for secondary findings. This is because, *at present,* the costs of hunting for secondary findings possibly do not meet the conditions of the duty of easy rescue – i.e., that the likely benefits significantly outweigh the costs, and that the costs are reasonably bearable. However, we suggest there would be value in conducting a research project that incorporates actively searching for a list of secondary findings in research participants undergoing genomic sequencing for other conditions.

According to most of the arguments we rejected above, researchers are *never* required to hunt for secondary findings. By contrast, one important upshot of our own argument is that even if genomic researchers do not currently have a duty to hunt for secondary findings, they may acquire such a duty in the near future as current barriers to hunting (such as the cost and effort required to do so) diminish. Accordingly, while human research ethics committees should not require researchers to factor searching for secondary findings into research protocols *at this point in time,* they will need to revisit the case for searching for secondary findings as the technology continues to develop. Given the current rate at which the technology in this field is improving, genomic researchers may soon be able to prevent serious harm at a cost that is comparatively small and reasonably bearable. Once these conditions are met, researchers *will* have a moral duty to search for secondary findings. The key question for the future is not whether genomic researchers have a moral duty to hunt for secondary findings*,* but rather how soon these conditions will obtain.

## Data Availability

Not applicable.

## Abbreviations

ACMG: American College of Medical Genetics and Genomics
FSGS: Focal segmental glomerulosclerosis
